# Carbon fibers coated with graphene reinforced TiAl alloy composite with high strength and toughness

**DOI:** 10.1038/s41598-018-20799-y

**Published:** 2018-02-05

**Authors:** Sen Cui, Chunxiang Cui, Jiaqi Xie, Shuangjin Liu, Jiejie Shi

**Affiliations:** Key Lab. for New Type of Functional Materials in Hebei Province, School of Material Science and Engineering, Hebei University of Technology, Tianjin, 300130 China

## Abstract

To meet the more rigorous requirement in aerospace industry, recent studies on strengthening and toughening TiAl alloys mostly focus on high Nb addition, which inevitably bring in an increasing of density. In this study, a carbon fibers coated with graphene reinforced TiAl alloy composite was fabricated by powder metallurgy, melt spun and vacuum melting. This composite got remarkable mechanical properties combined with a prominent density reduction. In contrast with pure TiAl ingots, this sample exhibits an average fracture strain from 16% up to 26.27%, and an average strength from 1801 MPa up to 2312 MPa. Thus, we can achieve a new method to fabricate this low-density, good mechanical performance TiAl composite which could bring in more opportunities for application in aerospace industry.

## Introduction

The strong need in aerospace such as gas turbine engines makes higher demands on the structural materials. In recent decades, TiAl alloys has gained great attention due to their low density, high specific strength and strength retention at elevated temperature^1^. In 2013, more than 40000 TiAl low pressure turbine (LPT) blades have been manufactured for the GEnx 1B (Boeing 787) and GEnx 2B (Boeing 747–8) turbine-engine application^2^. Now with the rapid development of aviation industry, TiAl alloys need to be furtherly strengthened and toughened. Guang Chen *et al*. has been obtained Ti-45Al-8Nb single crystals with 0° lamellar orientations by directional solidification, which exhibits a tensile ductility of 6.9% and a yield strength of 708 MPa^3^. W. C. Xu *et al*. has been manufactured a Ti-45Al-5Nb-0.8Mo-0.3Y alloys through hot extrusion and this TiAl-Nb-Mo alloy exhibits a yield strength of 812.2 MPa at room temperature, ultimate tensile strength of 343.5 MPa at 900 °C^4^. However, high Nb could severely increase the alloy density, which deviate from the lightweight purpose of aviation industry. Under the circumstance, fiber reinforcing could be a promising direction to strengthening and toughening TiAl alloys without the increasing of density. At this time, carbon fibers reinforced TiAl alloys were put forward in this study. The effects of carbon has been systematically studied in the previous studies, Thomas Klein *et al*. has been founding that C is enriched in the *α*_2_ phase, dissolved in the *γ* phase, but depleted in the *β*_*o*_ phase^5^. Carbon additions could improve the mechanical properties of TiAl alloys, particularly at elevated temperatures. Kawabata *et al*.^6^, Tian and Nemoto^7^ and Appel *et al*.^8–10^ reported on the positive effect of C on the tensile properties of intermetallic TiAl alloys. C can be acted as an efficient solid solution strengthener or form needle-shaped precipitates of perovskite-type carbides which are finely dispersed in the γ-phase^11^. Kawabata *et al*. observed a substantial increase in strength of C doped alloys due to solid solution hardening and precipitation of fine carbides^5^. Also, C is present in solid solution in the investigated alloys, which has been shown to effectively enhance creep performance^12,13^. Some fiber reinforced TiAl alloys were fabricated in recent years. A. Brunet *et al*. reported that alumina fibers coated with W, Y_2_O_3_ reinforced TiAl after the heat treatment (1 h at 1100 °C) provides an 80% increase in the mean tensile strength as compared with a monofilament composite without coating^14^. These fibers reinforcing alloys generally needs a metallic coating, such as W, Y_2_O_3_ coating on this alumina fibers via Physical Vapour Deposition (PVD) techniques. Besides, SiC fiber reinforced TiAl alloys fabricated by Luo X *et al*. got a C/Mo double coating via Chemical Vapor Deposition (CVD)^15^. In order to refine the wettability between the TiAl matrix and fibers, they usually introduce the reinforcements with the coating by CVD/PVD. In this study, however, interface bonding between carbon fibers/graphene and TiAl matrix was refined by several simple methods. First, the graphene coating on the surface of carbon fibers got a high surface energy. During the powder metallurgy, the graphene will react with the TiAl matrix and form TiC. Thus the interface could be refined by the *in-situ* reaction. Besides, the melt-spun process could make the TiAl rapidly quenched in the surface of carbon fibers. This rapidly quenched TiAl could be the interface between fibers and TiAl matrix in the vacuum melting process and refine the wettability.

In this study, carbon fibers coated with graphene were used as not only toughening fibers but also suppliers of strengthening ceramic particles such as TiC and Ti_2_AlC. Precipitated-phase strengthening mechanism and fiber toughening mechanism could reach a combination in this fabrication process. Besides, the reasonable sequence of several technologies is another vital factor in the remarkable reinforcement of mechanical properties. Continuous technologies including powder metallurgy, melt spun and two times’ vacuum melting could make the distribution of fibers in TiAl matrix more homogeneous, and improve wettability and density, respectively. This carbon fibers/graphene coating reinforced TiAl alloy composite (CFGRTAC) got an average fracture strain of 26.27%, which got a nearly 100% strain increase compared with pure TiAl alloys and a 20.1% density decrease compared with Ti-45Al-8Nb. We envisage that this carbon fibers coated with graphene reinforced TiAl alloy composite (CFGRTAC) could bring in a new orientation to fabricate composite materials in a simple and practicable way.

## Results

### Sintering bulk of carbon fibers/graphene reinforced TiAl alloys

**Figure 1:**
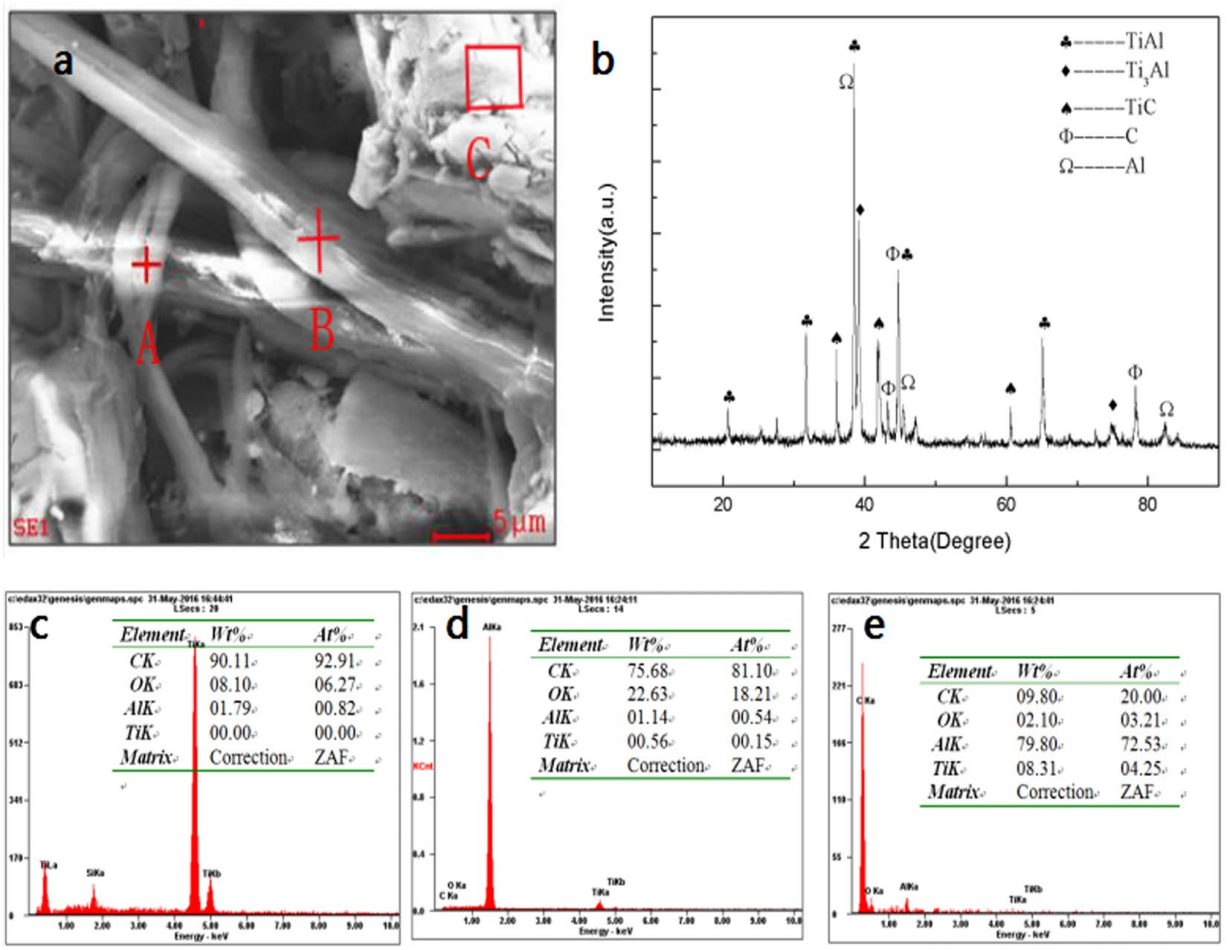
Microstructure and EDS patterns of carbon fibers coated with graphene reinforced TiAl alloys composite bulks. (**a**) SEM image and (**b**) XRD pattern, (**c**) EDS patterns of short rob in the marked area A, (**d**) EDS patterns of short rob in the marked area B and (**e**) EDS patterns of short rob in the marked area C of carbon fibers with graphene coating reinforced TiAl composite bulk (CFGCRTAC) sintered at 620 °C for 10 min.

Figure 1 shows the SEM image of composite bulks sintered at 620 °C for 10 min. As shown in Fig. 1a, rob-like carbon fibers just as A and B are distributed in the TiAl alloy matrix. Taking both the XRD spectra (Fig. 1b) and the EDS results (Fig. 1c–e) into account, it can be presumed that the point A, B and C in Fig. 1a are the carbon fiber A, carbon fiber B and TiAl alloy matrix, respectively. The phase composition of graphene/carbon fibers reinforced TiAl alloy composite are Ti_3_Al, TiAl, TiC and Ti_2_AlC. The newly formed Ti_2_AlC was synthesized by the *in situ* reactions as follows: Ti + C → TiC, TiC + TiAl → Ti_2_AlC^16^. As a matter of fact, in the terminology of metallography, Ti_2_AlC was formed by peritectic reaction between the TiAl melt and TiC particles^14^. During the sintering process, a part of carbon atoms reacts with Ti atoms in the metal matrix to form *in situ* TiC particles, and the rest carbon atoms are still distributed in the TiAl matrix.

### Carbon fibers/graphene reinforced TiAl alloys composite after the first remelting

**Figure 2:**
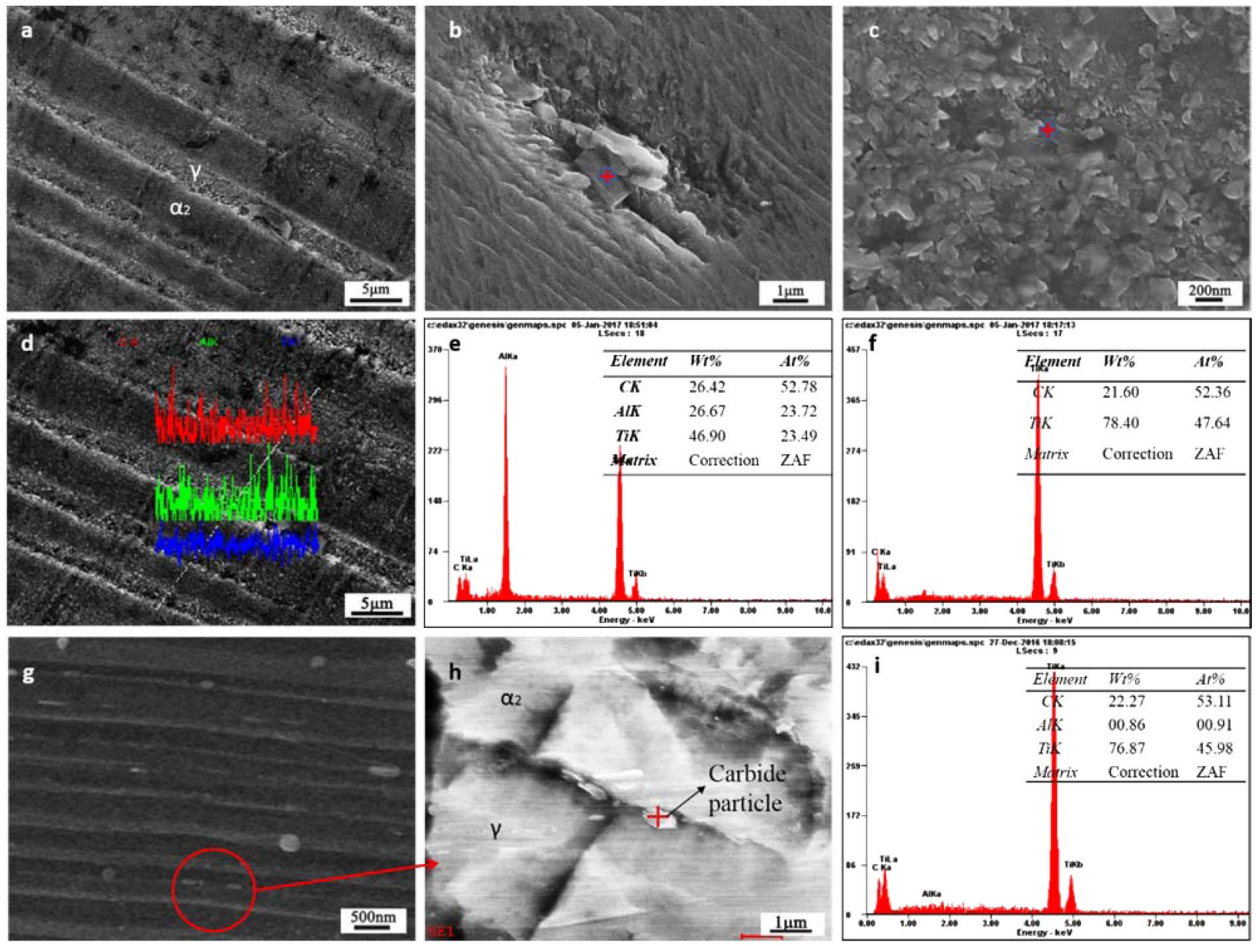
Microstructure and EDS patterns of CFGRTAC remelted for the first time. (**a**) SEM image of CFGCRTAC remelted; SEM images of (**b**) cuboid reinforcement, (**c**) spherical reinforcement, (**d**) EDS element line scanning patterns of the lamellar structure, EDS patterns of (**e**) cuboid reinforcement and (**f**) spherical reinforcement, SEM image of (**g**) the lamellar structure and (**h**) the magnification of interface in TiAl matrix; (**i**) EDS patterns of particle in the lamellar interface of TiAl matrix.

As shown in Fig. 2a, the interior morphology of the carbon fibers with graphene coating reinforced TiAl composite remelted was mainly in a form of lath trips. TiC was distributed in the matrix with many small spherical particles (Fig. 2c) and the Ti_2_AlC distributed in TiAl matrix as irregular particles (Fig. 2b). As shown in Fig. 2d, α_2_-Ti_3_Al and γ-TiAl are shown with the lamella structure. The increase of hardness is due to the result of carbon rich in α_2_ phase and the fine secondary carbide particles embedded in the γ phase^5,16,17^. Figure 2g,h show that small TiC particles are distributed in the interface of α_2_ phase and γ phase.

### Final carbon fibers/graphene reinforced TiAl alloys composite

**Figure 3:**
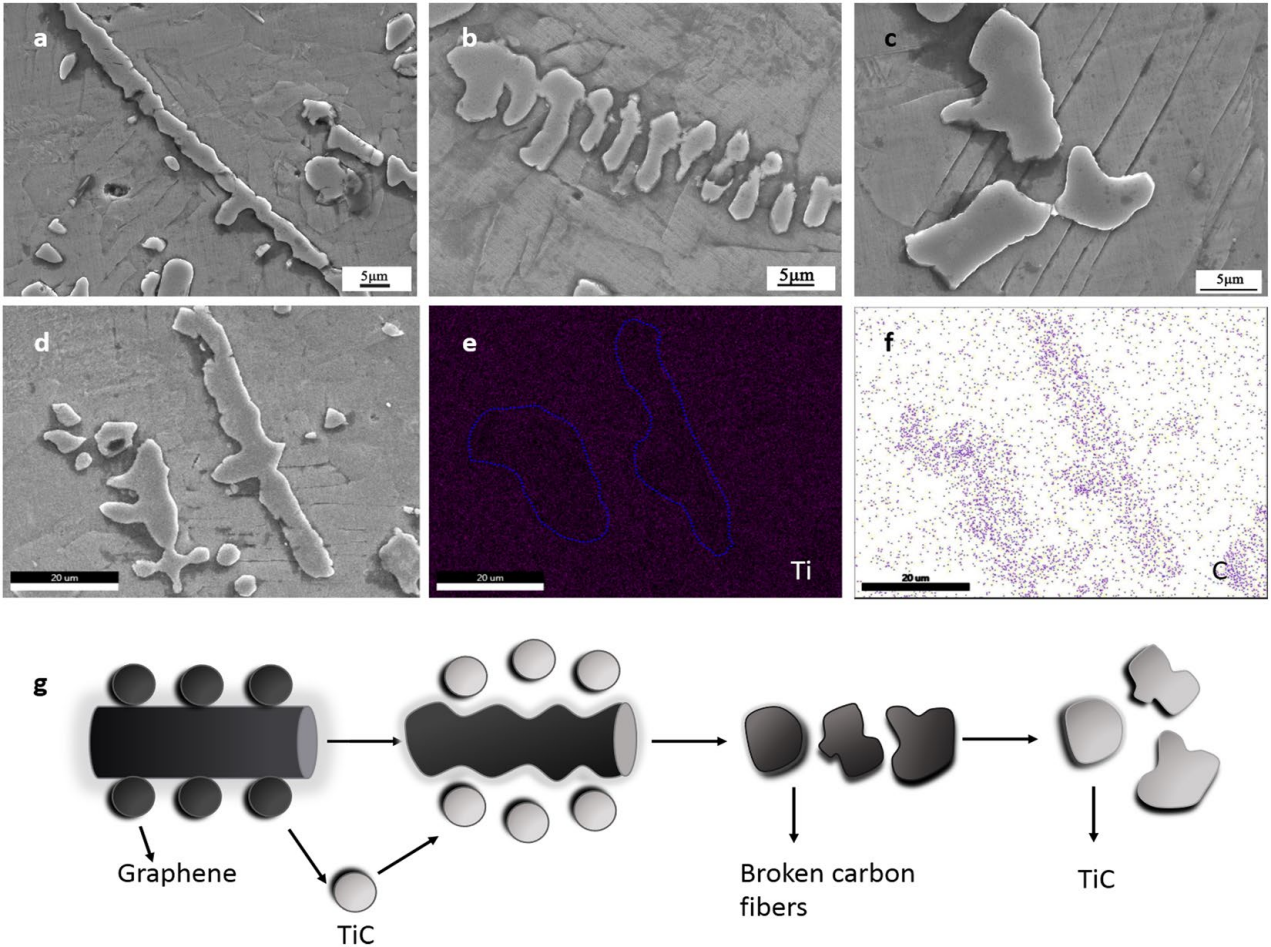
SEM images and map scan of CFGRTAC remelted twice. (**a**) Carbon fibers distribution in the TiAl matrix (**b**) a carbon fiber under reaction (**c**) reaction product TiC after the reaction of carbon fibers (**d**) complete carbon fibers (**e**,**f**) refer to the map scanning in Fig. 4d of Ti and C, respectively. (**g**) The evolution process model of carbon fibers in the TiAl matrix.

Figure 3a shows the microstructure of final CFGRTAC, as shown in Fig. 3a, a continuous carbon fiber was shown in the image. Figure 3b shows a broken carbon fiber during the reaction with TiAl matrix. Figure 3c is the final product after the reaction with matrix. Figure 3e,f refer to the map scanning in Fig. 3d of Ti and C, respectively. Figure 3d–f indicated that element C concentrate in the fiber regions and element Ti gets a relatively homogeneous distribution, however, it is obvious to observe that Ti got a less amount in the fiber regions. It can be conclude that the carbon fibers mostly reacted with Ti in the fiber surface, and the radical of the rod is still carbon.

The fiber evolution process could be summarized as the model in Fig. 3g. When getting a distribution in the TiAl matrix, the slight graphene coating got a higher surface energy and will react with TiAl first. Then the carbon fibers begin to participate in the reaction. The surface of graphene/carbon fibers tend to be zigzag, which means the carbon fibers/graphene start to form TiC and drift away. As the reaction goes on, the carbon robs gradually crack into fish-bone-shape, as shown in Fig. 3b. Finally, these broken carbon fibers exist as TiC and get a dispersive distribution in the TiAl matrix. Figure 3c shows the TiC particles intersect several lamellar layers in TiAl matrix.

### EBSD analysis of final CFGRTAC

**Figure 4:**
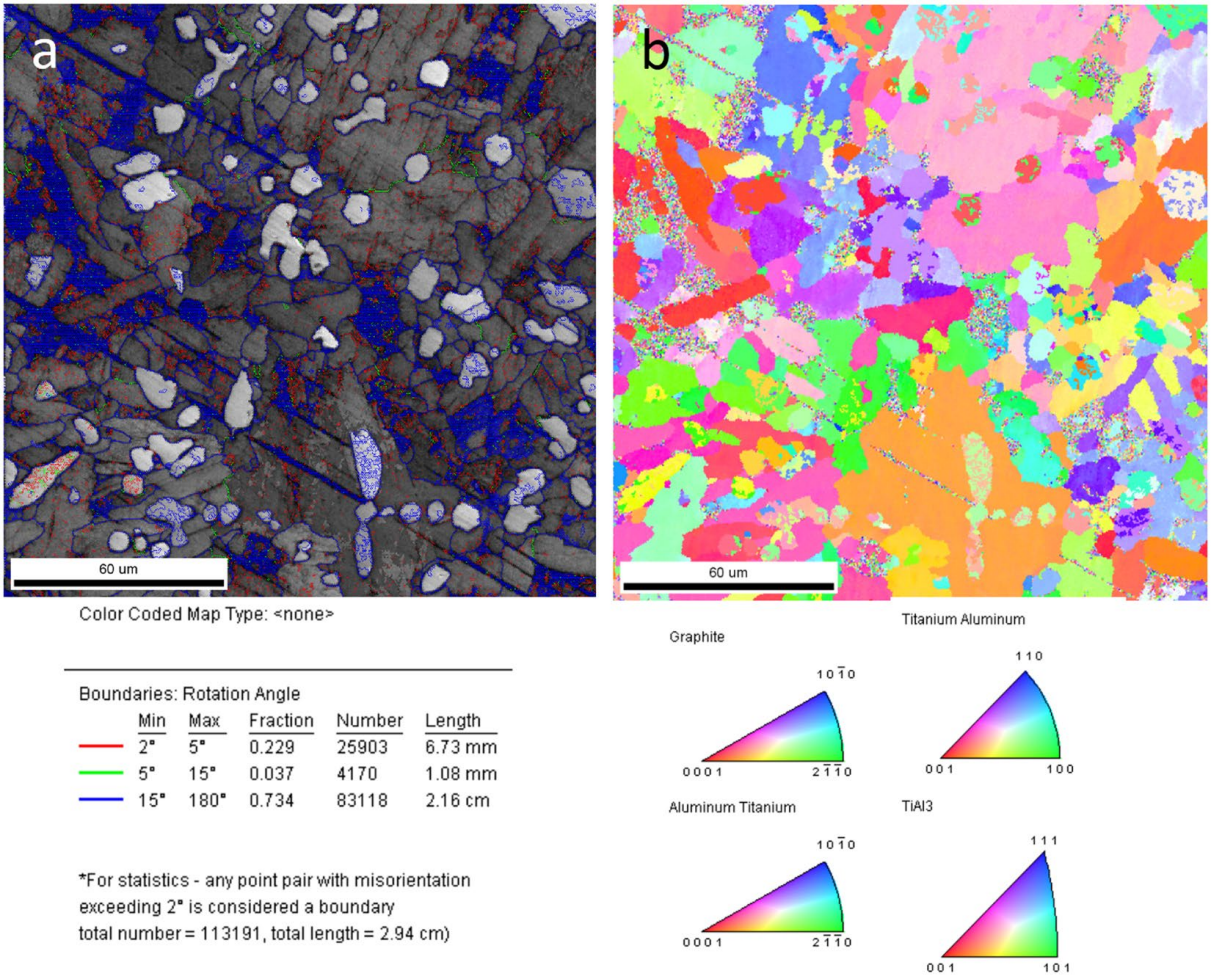
EBSD patterns of final CFGRTAC. (**a**) Microstructure and statistical regularity of rotation angles in grains boundaries. (**b**) Microstructure and phase distribution of CFGRTAC.

As shown in Fig. 4, as the carbon fibers and TiC particles distributed in the matrix, the grain size is smaller than 60 μm. With refining of grains, the volume fraction of high-angle boundaries is 73.4%. The refinement of grain size could enhance the strength and those high-angle boundaries could prevent crack deflection thereby improve the toughness.

### Microhardness test results and compression test results

**Figure 5:**
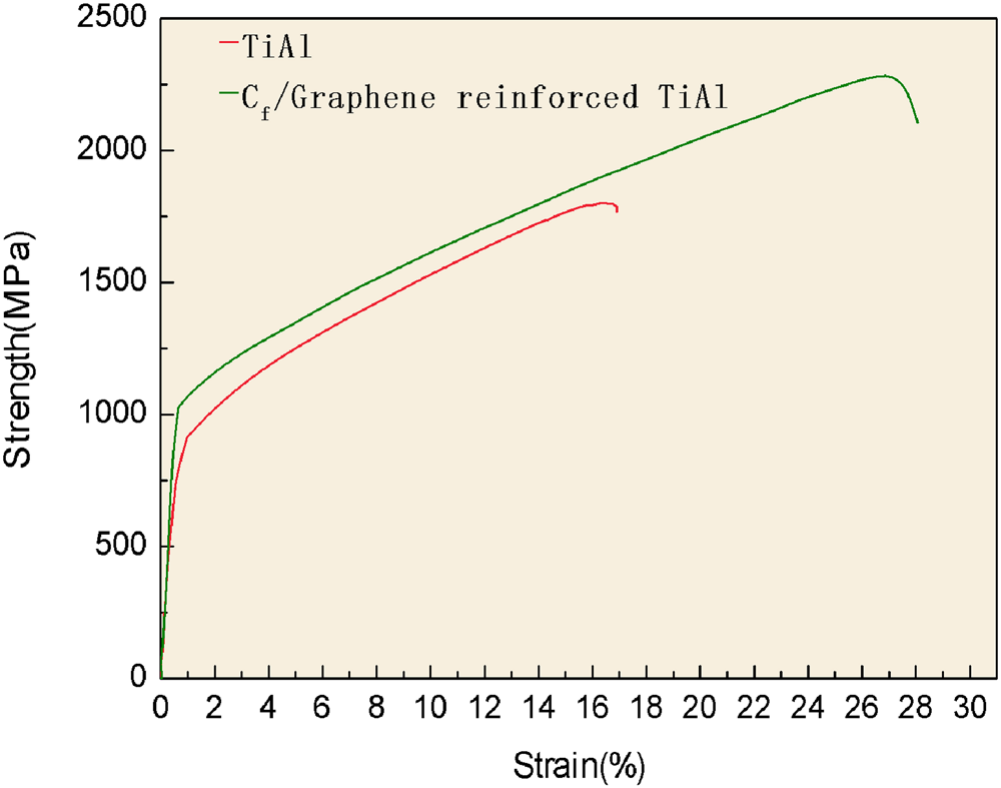
The compression curves of pure TiAl and final CFGRTAC.

As shown in Table 1. The microhardness of TiAl ingots, graphene reinforced TiAl alloy composite and graphene/carbon fibers reinforced TiAl alloy composite after twice remelting are 240 HV0.1, 216 HV0.1 and 426 HV0.1, respectively. The compression diagram of pure TiAl and the final CFGRTAC were shown in Fig. 5. We can see that this composite shows an average fracture strain from 17% up to 26.27% and average strength from 1801MPa to 2312 MPa. The strengthening and toughening mechanism of this CFGRTAC could be attributed to two aspects. On the one hand, it is by fine-grained strengthening. The combined effects of TiC and the unbroken carbon fibers provide the cores of the heterogeneous nucleation and refine the crystal. The second one is the increasement of interface debonding energy and the Bridging/Pull-out mechanism of carbon fiber toughening.

**Table 1:** Microhardness text of graphene/carbon fibers reinforced TiAl alloy composite.

Specimen	Microhardness (HV)
Normal TiAl ingots	216
Graphene reinforced TiAl alloy composite	240
Graphene/carbon fibers reinforced TiAl alloy composite	426

## Discussion

In this study, graphene reinforced TiAl exhibits a Vickers hardness of 240HV0.1. However, the carbon fibers/graphene reinforced TiAl alloys exhibit a much higher Vickers hardness of 426HV0.1. In the CFGRTAC, TiC and Ti_2_AlC particles play critical roles in the hardness’ improvement because of their own high hardness and the refine of the lamellar structure. As a α-phase stabilizing element, the great mass of carbon distributes in the dark lathes which are α_2_ phase, and the bright lathes are γ phase. Both α_2_ phase and γ phase could be harden by the C element^18,19^. As to the graphene reinforced TiAl alloys, the specimen got little increasement in Vickers hardness compared with the initial TiAl alloys. It is obvious that graphene reinforced TiAl alloys composite got similar phase composition with CFGRTAC. However, the simple vacuum melting with Ti and Al could not solve the problems in their bad interface bonding. It is attributed to the bad wettability between graphene and TiAl^15^. As a result, this graphene reinforced TiAl alloys composite couldn’t exhibits a good density to accomplish the further experiments.

**Figure 6:**
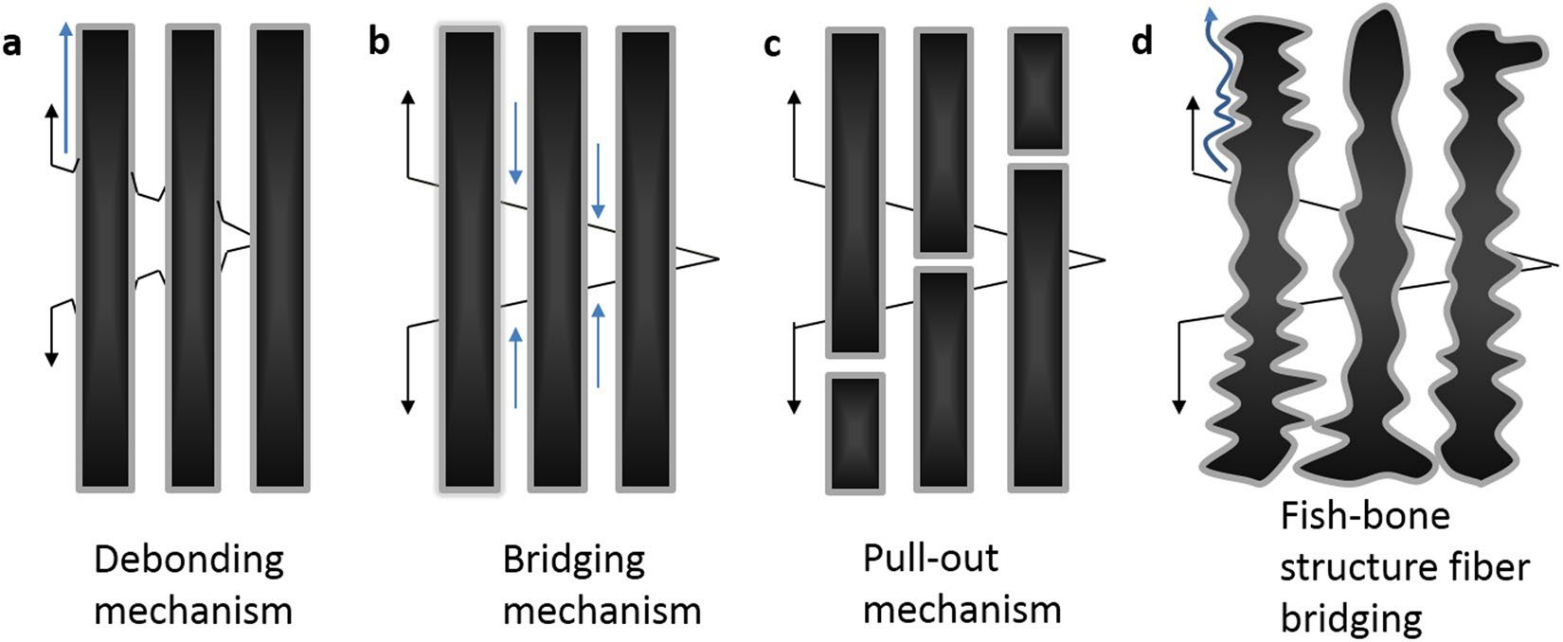
Sketch map of fiber toughening mechanism. (**a**) Interface debonding model, (**b**) bridging mechanism of intact fibers, (**c**) pull-out mechanism of broken carbon fibers (**d**) bridging model of fish-bone structure fibers.

In the fiber toughening alloys, Li^19^ has been verified that fiber volume fraction, interface shear stress, interface debonded energy, fiber Weibull modulus and fibers strength are all the impact elements of fiber-toughening materials. And when a crack generates from matrix, interface debonding is the first step before fibers fracture^20–22^. As shown in Fig. 6a, crack tips extension leads to the debonding of carbon fibers, which bring in many new microsurfaces. All the microsurfaces got a considerable surface energy in all. The energy consumption could postpone the crack propagation. In this study, as shown in Fig. 6, the fish-bone-like fibers get a zigzag surface which could enhance the distance of the crack deflection and enhance the interface debonding energy by increase the interface friction. Through this mechanism, the interface slip may cost much more energy, which could extend the slippage section. Besides, Lapin. J. *et al*. has been fabricated *in-situ* TiAl composite with (Ti, Nb)_2_AlC paricles and with the increasing content of C at a fixed content of Al, Nb, Mo and B, the ultimate compression strain shows a linearly increase^17^. Based on this situation, we can infer that the TiC, Ti_2_AlC in this study may also play a role in toughening TiAl alloys besides particle strengthening.

Figure 6b,c show the mechanism model when the crack starts to propagate furtherly after the fibers debonding. This process could be summarized into fiber bridging step (Fig. 6b) and fiber pull-out step (Fig. 6c). The crack propagation resistance K_r_ could be described as^23^

1 $${K}_{r}={K}_{m}+{\rm{\Delta }}{K}_{b}+{\rm{\Delta }}{K}_{p}$$

As shown in Eq. 1, K_m_ refers to matrix fracture toughness, ΔK_b_ denotes the resistance of bridging mechanism to the crack propagation, ΔK_p_ denotes the resistance of pull-out mechanism to the crack propagation. As applied stress increases and the crack started to extend, applied stress σ gradually increases but most fibers are still intact (Fig. 6b). Carbon fibers bring in a compressive stress to the crack which resists the crack propagation. In this step, fibers support the major stress to resist the applied stress. At this time, fiber-bridging play the leading role in arresting the crack propagation and ΔK_b_ is much higher than ΔK_p_. As shown in Fig. 6c. As the applied stress increases, the crack gets further expansion and surface of the crack tips start to expand. Many fibers start to slide or even crack. In this step, still carbon fibers fail to sustain the high stress, many fibers start to slide or even break, fibers slippage and fiber fracture furtherly resist the crack propagation by consuming much energy. At this time, ΔK_p_ increases rapidly to play the leading role in resisting the crack propagation.

Another nonnegligible toughening mechanism is the fish-bone-like fiber toughening. Unlike the normal fiber toughening alloys, the under reaction carbon fiber in this study exhibits a zigzag surface. As shown in Fig. 6d, this rough surface could increase the energy during the debonding, bridging, pull-out and break processes. As shown in Fig. 6a,d, the debonding displacement of fish-bone structure fiber is much larger than the normal fiber. Besides, this structure obviously got a higher fiction force. This high force of friction could make the dobonding and slippage more difficulty and toughening alloys in a more effective way than normal fiber toughening alloys composite.

## Conclusions

In summary, Graphene/carbon fibers reinforced TiAl alloy composite was fabricated by powder metallurgy, melt spun and remelting. The TiC and Ti_2_AlC particle were *in situ* synthesized during the fabrication processing. The graphene/carbon fiber reinforced TiAl alloy composite shows micronhardness of 426 HV0.1, average fracture strain of 26.27% and average strength of 2312 MPa.

This CFGRTAC was strengthened and toughened by several reasons. The melt spun process could refine interface between the alloys matrix and the graphene/carbon fibers. By introducing a rapid cooling TiAl coating in the carbon fiber/graphene, a better interface was built between fibers and matrix. On top of that, while fibers got a uniform dispersion in the TiAl matrix, dispersion strengthening was carried out by the TiC and Ti_2_AlC particles synthesized by *in situ* reaction. The other unreacted carbon fibers lay through many grains and multi-lamellar structure, which could enhance the interface debonding energy and make the crack tip deviated by the bridging toughening mechanism and pull-out toughening mechanism.

## Methods

### Specimen preparation

**Figure 7:**
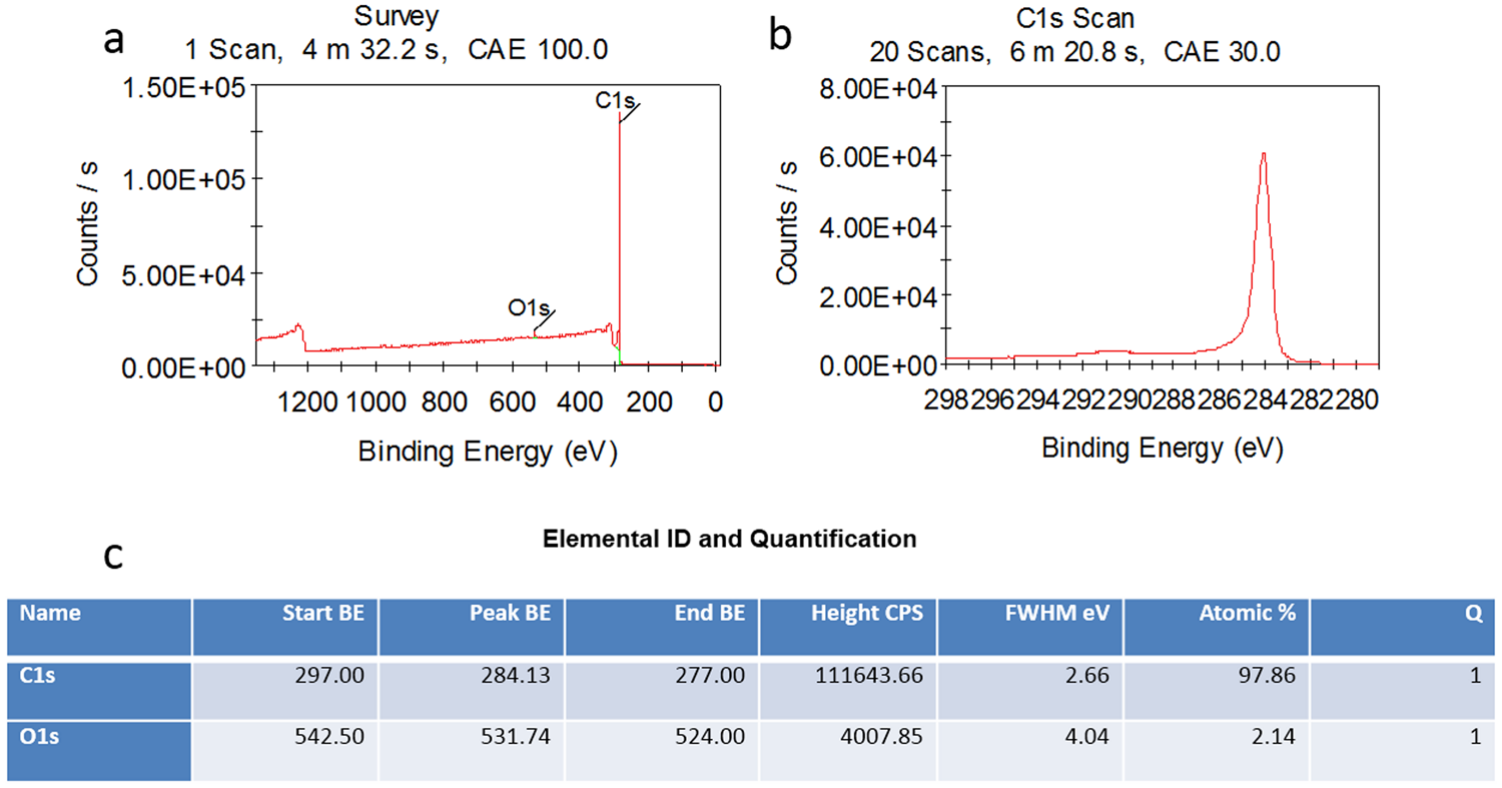
The XPS report of graphene powder. (**a**) XPS curves of all elements, (**b**) XPS curve of element carbon, (**c**) Elemental ID and Quantification.

**Figure 8:**
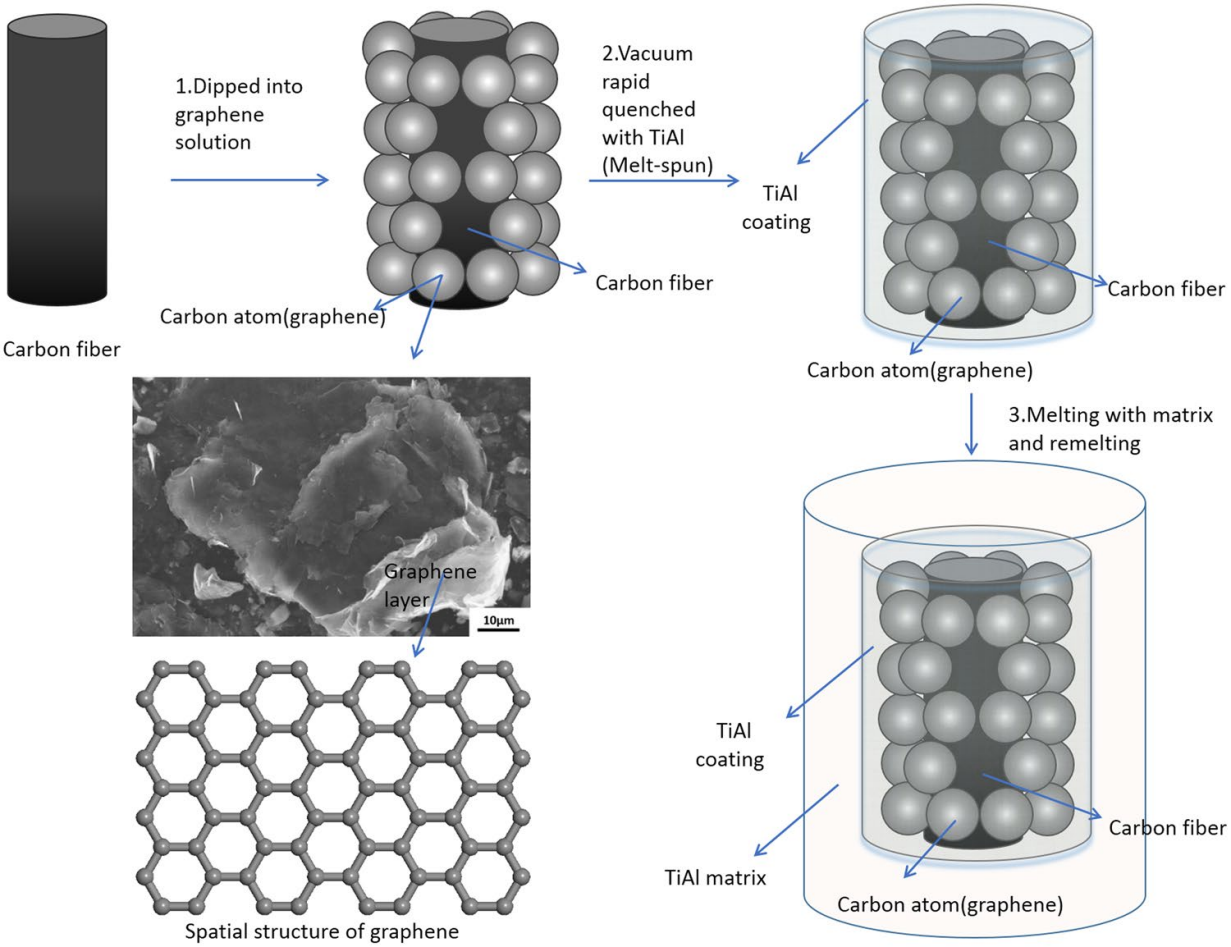
The technical processes and model of CFGRTAC.

Commercial pure Ti, Al ingots and Ti, Al powder, carbon fibers and graphene (solution) were used as raw materials in this study. Graphene was qualitative analyzed by XPS (Fig. 7) test. As the XPS results show, atomic proportion of element C and O are 97.86% and 2.14%, respectively. The existence of O is due to the stoving process of graphene solution. XPS curves show that element carbon got only one peak position in 284.13 eV, which accorded with the Carbon-Carbon Double Bond and Carbon-Carbon Single bond in 284.5 eV. The position of epoxy group (C-O, 286.4 eV), carbonyl group (C-O, 287.8 eV), or oxhydryl group (COOH, 289.0 eV) didn’t exist in this graphene. We can get the conclusion that carbon is the only element in this “graphene”. It is not oxidized in this study. The layer structure was observed by the SEM image in Fig. 8. Ti-50Al alloy was fabricated by Ti and Al ingots in ZGX-5 vacuum induction melting furnace. Graphene was coated on the surface of rod-like carbon fibers (about 2 mm in length, 8–10 μm in diameter). Ti, Al powder and carbon fibers with graphene coating were sintered by GL-100 vacuum-sintering furnace in 620 °C for 10 min in the atmosphere of argon to fabricate the initial bulks as the raw materials in the next procedure. Those graphene/carbon fibers-TiAl composite bulks were melt-spun in the LZK-12A spun furnace to get composite ribbons, and then these composite ribbons and TiAl alloy were remelted in ZGX-5 vacuum induction melting furnace to get the carbon fibers with graphene coating reinforced TiAl composite(Ti-50Al-3C at.%). Finally, in order to improve the density, a small bulk was cut from this TiAl composite and remelted into an Ø20 × 60 mm CFGRTAC specimen by MZG high frequency vacuum melting furnace. The model of technology flow diagram was shown in Fig. 8. At the same time, a graphene reinforced TiAl alloy composite was fabricated by ZGX-5 vacuum induction melting furnace as the comparison specimen. Vickers hardness test was carried out by SHIMADZU HMV-2 Vickers hardness tester under a load value of 980.7 mN, load time of 10 s. The microhardness test was tested ten times for each samples. The average numbers were used as the final results. Compression specimens with an approximate size of Ø2 × 4 mm were cut from those samples. Three samples were tested and the average value was regarded as a final value. And the compression tests were carried out at room temperature with an initial strain rate of ε′ = 2.5 × 10^−4^ S^−1^.

### Microstructure characterization

The field emission scanning electron microscopy (FESEM S-4800, Hitachi, Japan) equipped with energy-dispersive spectrometry (EDS) were used to characterize the microstructure of the specimens. The microstructures of the polished specimens were etched with the agent comprising 85 ml distilled water, 10 ml HNO_3_ and 5 ml HF.

### Phase analysis

The X-ray diffraction (XRD Bruker, German) was used to identify the phases of the specimens.
